# Approaches to discontinuing efalizumab: an open-label study of therapies for managing inflammatory recurrence

**DOI:** 10.1186/1471-5945-6-9

**Published:** 2006-10-26

**Authors:** Kim A Papp, Darryl Toth, Les Rosoph

**Affiliations:** 1 Probity Medical Research, Waterloo, and University of Western Ontario, Ontario, Canada; 2 Probity Medical Research, Windsor, Ontario, Canada; 3 Probity Medical Research, North Bay, Ontario, Canada

## Abstract

**Background:**

Efalizumab is a humanised recombinant monoclonal IgG1 antibody for the treatment of moderate-to-severe plaque psoriasis. When treatment discontinuation is necessary, however, some patients may experience inflammatory recurrence of the disease, which can progress to rebound if untreated. This analysis evaluated approaches for managing inflammatory recurrence after discontinuation of efalizumab.

**Methods:**

An open-label, multicentre, investigational study was performed in 41 patients with moderate-to-severe plaque psoriasis who had recently completed clinical studies with efalizumab and had developed signs of inflammatory recurrence following abrupt cessation of treatment. Patients were assigned by the attending physicians to receive one of five standardised alternative systemic psoriasis treatment regimens for 12 weeks. Efficacy of the different therapy options was assessed using the physician's global assessment (PGA) of change over time.

**Results:**

More favourable PGA responses were observed in patients changing to cyclosporin (PGA of 'good', 'excellent' or 'cleared': 7/10 patients, 70.0%) or methotrexate (9/20, 45.0%), compared with those receiving systemic corticosteroids (2/8, 25.0%), retinoids (0/1, 0.0%) or combined corticosteroids plus methotrexate (0/2, 0.0%). While the majority (77.8%) of patients showed inflammatory morphology at baseline, following 12 weeks of the alternative therapies the overall prevalence of inflammatory disease was decreased to 19.2%.

**Conclusion:**

Inflammatory recurrence after discontinuation of efalizumab therapy is a manageable event, with a number of therapies and approaches available to physicians, including short courses of cyclosporin or methotrexate.

## Background

Psoriasis is an inflammatory skin disorder that affects approximately 2–3% of the population [1] and has a profound impact on quality of life, equivalent to that of other major diseases [2]. Patients with the disease present with well-defined, thickened erythematous patches, typically covered with a silver scale, and the condition is characterised by epidermal hyperplasia, dermal angiogenesis, infiltration of activated T-cells and increased cytokine levels [3]. Chronic psoriasis is a condition requiring long-term medication. Systemic therapies are required by patients with moderate-to-severe disease; a variety of systemic therapies are available, but many of the current agents have serious side-effects that limit long-term administration [4,5].

The aetiology and pathology of psoriasis are not well understood, but it has been established that T-cells are centrally involved in its development [3]. Recent understanding of the inflammatory pathways in psoriasis has led to the development and use of new biologic agents to treat the condition. One such biologic therapy, efalizumab, is a humanised recombinant monoclonal IgG1 antibody. Efalizumab binds to the alpha-subunit, CD11a, of the T-cell adhesion molecule, leukocyte function-associated antigen-1 (LFA-1), preventing binding with its ligand, intercellular adhesion molecule-1 (ICAM-1), on target cells. This action blocks several T-cell processes important in the pathogenesis of psoriasis, including T-cell activation, T-cell trafficking from the circulation into the skin, and T-cell reactivation in the dermis and epidermis [3,6,7].

Several large clinical studies have established the safety and efficacy of efalizumab during extended treatment of patients with moderate-to-severe chronic plaque psoriasis [8-10]. There are occasions, however, such as during pregnancy or following vaccination or adverse events, when patients have to stop efalizumab treatment. When treatment was stopped abruptly in controlled studies, relapse of psoriasis was reported, with exacerbation and new morphology of psoriasis (see Table 1 for definitions of terms such as 'relapse', 'rebound' and 'flare') [11]. There is currently little evidence-based medicine to guide management and treatment of patients after discontinuation of efalizumab. This study (protocol # IMP25180) evaluated five regimens of standard systemic treatments for psoriasis that have been in use for a number of years, with a view to identifying appropriate therapy for the treatment of 'inflammatory recurrence' following discontinuation of efalizumab. The term 'inflammatory recurrence' was used in this study to cover two scenarios that may prompt re-initiation of treatment in routine clinical practice: (1) patients experience worsening of psoriasis soon after discontinuing treatment (within 2–3 months) that is not considered to reflect worsening due to the natural course of the disease, but that has not worsened sufficiently to constitute a rebound; or (2) patients have discontinued a psoriasis treatment due to an inflammatory disease flare but, following discontinuation, require treatment to prevent a rebound. For both these scenarios, it is important to re-initiate treatment promptly to prevent a rebound of psoriasis, which has been reported in approximately 5% of patients following cessation of efalizumab treatment[12] However, it should be noted that the second scenario is very uncommon – in phase III studies of efalizumab only 0.6% of efalizumab-treated patients discontinued treatment due to recurrence of psoriasis[13].

This study was intended to mimic current practice and hence a naturalistic approach, in which the physician chose the most appropriate therapy, was pursued. The main objective was to provide guidance for appropriate management of inflammatory recurrence before its progression to rebound.

## Methods

This was an open-label, investigational study carried out in nine centres on patients with moderate-to-severe plaque psoriasis. The study was carried out according to Good Clinical Practice guidelines and the Declaration of Helsinki; the protocol was approved by Research Review Board, Inc., and all patients provided signed informed consent. Patients were enrolled in the study if: (1) they had worsening of psoriasis within 2 months of discontinuation from efalizumab treatment in other studies, which in the opinion of the investigator had not worsened sufficiently to constitute a rebound but that required re-initiation of treatment or, (2) had previously discontinued an efalizumab study due to an inflammatory disease flare. To cover both these scenarios, the term 'inflammatory recurrence' has been used. Patients were included if the recurrence was related to the disease previously treated with efalizumab and were excluded if the recurrence was considered to be part of the natural disease progression, which occurs more slowly (i.e. a relapse; see Table 1 for the EMEA definition of 'relapse').

Eligible patients received 12 weeks of systemic psoriasis therapy during the study as soon as inflammatory recurrence had been identified by the investigator. In order for the study to closely reflect routine clinical practice, attending physicians were free to judge which patients had worsening of disease that had not progressed to rebound and subsequently to choose the therapy they considered most appropriate for each individual patient from five pre-defined regimens. Physicians could switch among these therapies during the course of the study if it was deemed necessary. The standard approved psoriasis therapy regimens were:

• cyclosporin: initial dose 4.0–5.1 mg/kg/day until clinical improvement (as judged by the investigator), followed by a 50% reduction in dose every 2 weeks

• retinoids: initial dose 25–50 mg/day until clinical improvement (as judged by the investigator), followed by a 50% reduction in dose continuing for 8 weeks, at which time therapy was stopped

• corticosteroids: initial dose 0.25–0.5 mg/kg/day until clinical improvement (as judged by the investigator), followed by a 50% reduction in dose every 2 weeks

• methotrexate: initial dose 20–25 mg/week until clinical improvement (as judged by the investigator), followed by a 25% reduction in dose every 2 weeks

• combined therapy: systemic corticosteroids plus methotrexate, utilising both of the above regimens in combination.

The physician's global assessment (PGA) is a simple and quick-to-use assessment of clinical status in patients with psoriasis. The PGA is familiar to physicians and is the standard measure of disease severity in current clinical practice. Therefore, PGA of change was considered to be the simplest and most appropriate measure to assess treatment efficacy in the present study. PGA of change was categorised according to seven ratings: 'clear' (100% improvement from baseline), 'excellent' (75–99%), 'good' (50–74%), 'fair' (25–49%), 'slight' (1–24%), 'unchanged' (no change in clinical signs and symptoms from baseline) and 'worse' (deterioration of clinical signs and symptoms from baseline). The disease was classified according to the morphology as inflammatory, plaque, papular/pustular, erythrodermic or inverse psoriasis and individual patients could have more than one type. The proportion of patients with each type was evaluated at the start and end of treatment.

This was a pilot investigation designed to collect information that would help plan future management strategies for patients who experience inflammatory recurrence after discontinuing efalizumab. There was no randomisation, all patients were analysed as treated and no formal statistical analysis was carried out. Owing to the need for treatment to control psoriasis, some patients were already allocated to one of the study drugs at the time of enrolment into the study. For these patients, baseline data were collected retrospectively, while data were collected prospectively for patients who were prescribed their initial psoriasis treatment at the time of enrolment. As the results were qualitatively similar for the two subgroups, the results are presented only for the two subgroups analysed together.

## Results

A total of 41 patients were enrolled in the study (24 retrospectively and 17 prospectively), and their demographic details are summarised in Table 2 according to the systemic treatment initially prescribed by the attending physician. The age and gender profiles were similar across treatment groups. The systemic treatment most commonly prescribed was methotrexate, which was given as a single therapy to 20 patients; this was followed by cyclosporin, given to 10 patients, and corticosteroids, given to eight patients. For the majority of the patients in each treatment group, administration of one or more of the other systemic treatments was possible.

There were five patients in total who discontinued treatment before completing 12 weeks of the study. Three of these patients were from the methotrexate group: two discontinued due to patient decision and one was lost to follow-up; of the remaining two patients who discontinued prematurely, one discontinued from the cyclosporin group due to lack of efficacy, and one discontinued from the combined methotrexate plus corticosteroid group due to patient decision.

### Assessment of PGA response

PGA for changes in disease was rated on a seven-point categorical scale from improvement resulting in clear of disease to worsening of disease. The proportions of patients categorised as 'good', 'excellent' or 'cleared' are shown in Table 3. The results in Table 3 are summarized according to the initial treatment prescribed to manage inflammatory recurrence (referred to as 'first treatment'), as well as according to the treatment that the patient was receiving at the end of the study (referred to as the 'last treatment'). This categorization of the results was designed to account for six patients who were switched between treatments during the study; similar results were observed when PGA was summarised by the first or last treatment. Cyclosporin provided the most favourable response, with a 'good', 'excellent' or 'cleared' improvement recorded for 7/10 (70.0%) patients who started on the drug. A 'good', 'excellent' or 'cleared' rating was also recorded for 9/20 (45.0%) patients who started on treatment with methotrexate.

### Changes in psoriasis morphology

Psoriasis morphology at the start and end of treatment is summarised in Table 4, according to the last treatment prescribed. As morphology of disease was not available for all patients, the data are shown as patients with a particular morphology as a proportion of the number of patients with available data at baseline. For all treatment groups combined, 21/27 (77.8%) patients with baseline morphology data had inflammatory psoriasis at entry. After treatment with a systemic psoriasis therapy, the prevalence of inflammatory disease was reduced to 19.2% of the patients. Methotrexate appeared to provide the best response for inflammatory psoriasis: the prevalence was reduced from 84.6% at baseline to just 8.3% at the end of treatment. Methotrexate also appeared to reduce the prevalence of papular/pustular psoriasis more than the other treatments.

The adverse event profile associated with each of the medications has been well documented, and the safety results were consistent with the known profiles. Across all treatment groups, 14 patients (34.1%) had at least one adverse event. Adverse events were considered to be possibly or probably related to treatment in eight patients (19.5%). During the study, there were no deaths, serious adverse events, or discontinuations due to adverse events.

## Discussion

Efalizumab has been developed for long-term treatment of psoriasis, and clinical studies have shown increasing response with longer treatment duration. However, on discontinuation of treatment there have been reports of rebound of psoriasis symptoms, mainly in non-responding patients [11,14]. Rebound can occur with other psoriasis medications as was shown many years ago after stopping systemic corticosteroids [15] as well as cyclosporin [16,17]. If alternative therapies are instituted immediately when inflammatory recurrence is observed, development of rebound may be prevented. This analysis was a preliminary exploration of appropriate alternative therapeutic regimens for control of inflammatory disease recurrence in patients discontinuing efalizumab, such that progression to rebound can be avoided. As an exploratory study, there are a number of inherent limitations that should be accounted for when interpreting the results, including the small number of patients included in each of the treatment groups. It should also be noted that, due to the need for treatment to control psoriasis, some patients were already allocated to one of the study drugs at the time of enrolment into the study. Therefore, baseline data were collected retrospectively for these patients.

In clinical trials, the Psoriasis Area and Severity Index (PASI) is the most commonly used measure of disease severity, but in a clinical setting it is difficult to use due to its complexity. The PGA, which is more simple and quick to use compared with the PASI is, therefore, the more commonly used assessment in current clinical practice. In a placebo-controlled clinical study of efalizumab for the treatment of patients with moderate-to-severe plaque psoriasis, close agreement was found between the PASI and PGA when both were used to measure improvement in psoriasis [18]. In addition, a double-blind trial directly compared ratings with the PASI, PGA and a National Psoriasis Foundation scoring system and found a strong concordance between them [19]. Therefore, PGA was considered to be the simplest and most appropriate measure for use in the present study.

The results from the present analysis indicated that methotrexate and cyclosporin were effective in alleviating the symptoms of recurrence of psoriasis. Systemic corticosteroids and retinoids appeared less effective in treating inflammatory recurrence, but the small number of patients means that further studies with these therapies are still necessary.

## Conclusion

The results from this study indicate that inflammatory recurrence after discontinuation of efalizumab therapy is a manageable event. In order to prevent progression to established rebound, a number of therapies and approaches are available to physicians, including short courses of cyclosporin or methotrexate. Further larger-scale studies are required to confirm the present results.

## Competing interests

KAP, DT and LR have received consultancy honoraria from Serono.

## Authors' contributions

KAP, DT and LR all contributed to the design and conduct of the study, and to the analysis and interpretation of the results. All authors read and approved the final manuscript.

## Pre-publication history

The pre-publication history for this paper can be accessed here:



## Figures and Tables

**Table 1:** Definitions of commonly-used terminology used by the US National Psoriasis Foundation (NPF), the European Medicines Agency (EMEA) and this study.

**Term**	**NPF [20]**	**EMEA [21]**	**This study**
**During treatment**			
Flare	Typical or unusual worsening of disease during treatment and/or the occurrence of new psoriasis morphologies.	Not defined.	Not used
**Following treatment discontinuation**			
Recurrence	Not defined	The EMEA recommend the use of recurrence as an endpoint in long-terms studies provided that it is clearly defined.	The term 'inflammatory recurrence' includes (a) patients with worsening of psoriasis within 2 months of discontinuation from efalizumab treatment to a level less severe than a rebound and (2) patients who have discontinued a psoriasis treatment due to an inflammatory disease flare but, following discontinuation, require treatment to prevent a rebound.
Relapse	Loss of 50% of PASI improvement from baseline in patients who achieve a clinically meaningful response (≥ 50% improvement in PASI score from baseline).	A reduction of >50% in PASI from the achieved maximal improvement in PASI score. Subjective alternative: a relapse of psoriasis necessitating the re-initiation of treatment. A simple worsening of psoriasis beyond 2 months of therapy may represent the natural course of the disease (relapse) rather than a rebound associated with drug.	Not used
Rebound	A PASI of 125% of baseline or new generalized pustular, erythrodermic, or more inflammatory psoriasis occurring within 3 months of stopping therapy. Worsening occurring after 3 months of therapy may represent the natural course of the disease rather than a rebound associated with the drug.	Worsening of psoriasis over baseline value (e.g. PASI>125%) or new pustular, erythrodermic or more inflammatory psoriasis occurring within 2 months of stopping therapy. A 2-month boundary separating relapse and rebound is drawn on theoretical grounds and is more or less arbitrary.	Not used

PASI, Psoriasis Area and Severity Index

**Table 2:** Patient demographics at baseline, according to systemic therapy prescribed as first treatment.

	**Methotrexate (N = 20)**	**Cyclosporin (N = 10)**	**Corticosteroids (N = 8)**	**Combined^**a **^(N = 2)**	**Retinoids (N = 1)**	**All patients (N = 41)**
**Age (years)**						
Mean ± SD	49 ± 10	43 ± 12	44 ± 11	47 ± 3	41	46 ± 11
Range	28–67	20–58	20–60	45–49	-	20–67
**Gender (n (%))**						
Male	13 (65.0)	5 (50.0)	5 (62.5)	1 (50.0)	1 (100)	25 (61.0)
Female	7 (35.0)	5 (50.0)	3 (37.5)	1 (50.0)	0	16 (39.0)
**Ethnicity (n (%))**						
Caucasian	15 (75.0)	10 (100)	8 (100)	2 (100)	1 (100)	36 (87.8)
Asian	2 (10.0)	0	0	0	0	2 (4.9)
Other	3 (15.0)	0	0	0	0	3 (7.3)

^a^Combined systemic corticosteroids plus methotrexate.

**Table 3:** Patients with a PGA change^a ^of 'good', 'excellent' or 'cleared', according to first treatment (assigned at baseline) and last treatment (the treatment that was being received at Week 12)*.

	**Methotrexate**	**Cyclosporin**	**Corticosteroids**	**Combined**^**b**^	**Retinoids**	**All patients**
**First treatment (n (%))**	9/20 (45.0)	7/10 (70.0)	2/8 (25.0)	0/2 (0.0)	0/1 (0.0)	18/41 (43.9)
**Last treatment (n (%))**	9/18 (50.0)	9/12 (75.0)	4/8 (50.0)	1/2 (50.0)	0/1 (0.0)	23/41 (56.1)

* At the investigators' discretion, six patients were switched between treatments during the study.

^a^Change from baseline.

^b^Combined systemic corticosteroids plus methotrexate.

PGA, physician's global assessment.

**Table 4:** Disease morphology at start of treatment (baseline) and at Week 12 or withdrawal (endpoint), by last treatment prescribed^a^.

	**Methotrexate (N = 13)**	**Cyclosporine (N = 8)**	**Corticosteroids (N = 4)**	**Combined^**b **^(N = 2)**	**All patients (N = 27)**
**Inflammatory (n (%))**					
Baseline	11/13 (84.6)	6/8 (75.0)	2/4 (50.0)	2/2 (100)	21/27 (77.8)
Week 12	1/12^c ^(8.3)	3/8 (37.5)	0/4 (0.0)	1/2 (50.0)	5/26^c ^(19.2)
**Plaque (n (%))**					
Baseline	7/13 (53.8)	7/8 (87.5)	4/4 (100)	2/2 (100)	23/27 (85.2)
Week 12	12/12^c ^(100)	7/8 (87.5)	4/4 (100)	2/2 (100)	25/26^c ^(96.2)
**Papular/pustular (n (%))**					
Baseline	8/13 (61.5)	3/8 (37.5)	1/4 (25.0)	1/2 (50.0)	13/27 (48.1)
Week 12	2/12^c ^(16.7)	3/8 (37.5)	0/4 (0)	1/2 (50.0)	6/26^c ^(23.1)
**Inverse (n (%))**					
Baseline	1/13 (7.7)	0/8 (0.0)	1/4 (25.0)	0/2 (0.0)	2/27 (7.4)
Week 12	1/12^c ^(8.3)	0/8 (0.0)	0/4 (0.0)	0/2 (0.0)	1/26^c ^(3.8)
**Erythrodermic (n (%))**					
Baseline	0/13 (0.0)	1/8 (12.5)	0/4 (0.0)	0/2 (0.0)	1/27 (3.7)
Week 12	0/12^c ^(0.0)	0/8 (0.0)	0/4 (0.0)	0/2 (0.0)	0/26^c ^(0.0)

^a^Values shown are the numbers of patients with the type of morphology as a proportion of the number of patients with available data at baseline. At the investigators' discretion, six patients were switched between treatments during the study. No patient received retinoids at Week 12.

^b^Combined systemic corticosteroids plus methotrexate.

^c^One patient withdrew from the study.
